# Development of stimuli-responsive nano-based pesticides: emerging opportunities for agriculture

**DOI:** 10.1186/s12951-019-0533-8

**Published:** 2019-09-21

**Authors:** Marcela Candido Camara, Estefânia Vangelie Ramos Campos, Renata Aparecida Monteiro, Anderson do Espirito Santo Pereira, Patrícia Luiza de Freitas Proença, Leonardo Fernandes Fraceto

**Affiliations:** 1 São Paulo State University – UNESP, Institute of Science and Technology, Sorocaba, SP Brazil; 2 Human and Natural Sciences Center, Federal University of ABC, Santo André, SP Brazil

**Keywords:** Nanopesticides, Triggered release, Smart delivery, Biotic stress, Abiotic stress, Sustainable agriculture

## Abstract

Pesticides and fertilizers are widely used to enhance agriculture yields, although the fraction of the pesticides applied in the field that reaches the targets is less than 0.1%. Such indiscriminate use of chemical pesticides is disadvantageous due to the cost implications and increasing human health and environmental concerns. In recent years, the utilization of nanotechnology to create novel formulations has shown great potential for diminishing the indiscriminate use of pesticides and providing environmentally safer alternatives. Smart nano-based pesticides are designed to efficiently delivery sufficient amounts of active ingredients in response to biotic and/or abiotic stressors that act as triggers, employing targeted and controlled release mechanisms. This review discusses the current status of stimuli-responsive release systems with potential to be used in agriculture, highlighting the challenges and drawbacks that need to be overcome in order to accelerate the global commercialization of smart nanopesticides.

## Introduction

One of the main current challenges facing global agriculture is the need to control and reduce the intensive use of agrochemicals such as herbicides, fungicides, and insecticides. It is well known that these substances can have adverse environmental and social impacts, as well as cause resistance in target organisms [1]. Furthermore, large amounts of pesticides are lost during application, due to volatilization, degradation, and photolysis, with less than 0.1% of applied pesticides effectively acting against the target organisms [2]. Nanotechnology seems to offer a way to mitigate the harmful effects of pesticides on the environment and human health, since it can provide systems enabling the controlled release of active compounds, thus increasing the efficiency and safety of products, while reducing the quantities required in field applications.

**Fig. 1:**
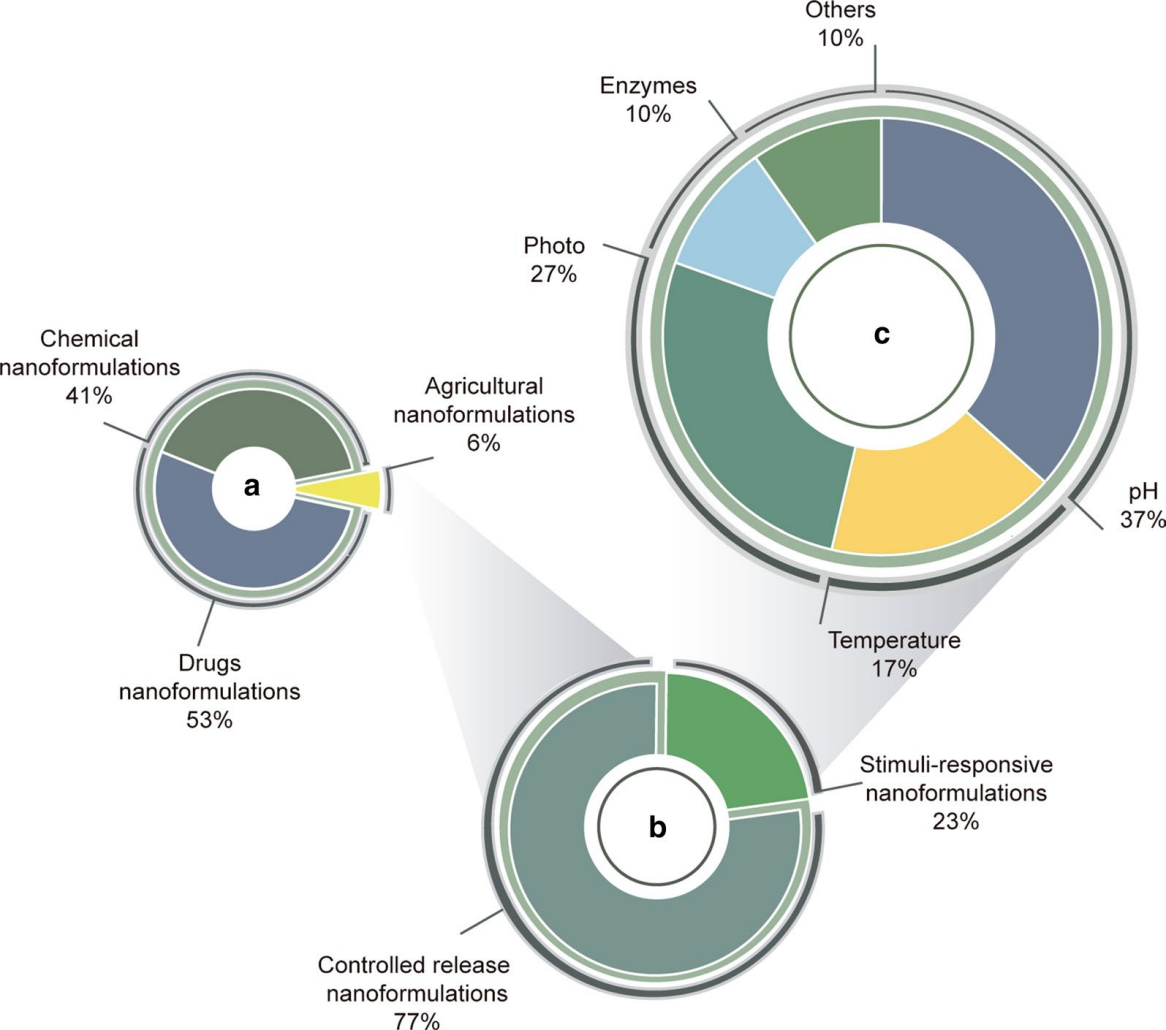
Survey of published research available in the Scopus database for the period 2009–2019. The search was performed using the following keywords: **a** nanoformulations; **b** controlled release, stimuli-responsive nanoformulations; and **c** pH, temperature, photo, enzymes, redox, magnetic field-responsive (search performed in June 2019)

Controlled release systems are emerging technologies that have attracted global commercial and scientific interest in recent years. Such systems are used in areas including medicine, cosmetics, engineering, food, and agriculture [3]. Considering their use in the agricultural and environmental sector, a survey using the Scopus database (considering the period from 2009 to 2019) showed that only 6% of research concerning nanoformulations was related to the agroindustry field, of which 77% concerned controlled release nanoformulations, while only 23% involved stimuli-responsive materials (Fig. 1). Considering stimuli-responsive materials, pH was the most widely studied stimulus (37%), followed by photo and thermal stimuli (27% and 17%, respectively). Enzymes, redox, and other types of stimuli together accounted for 20% of the studies (Fig. 1).

The commercial trends of micro- and nano-formulations were investigated by analyzing patent data. A search using specialized patent databases (Questel Orbit and Espacenet), employing the IPC classification A01N25/28 (micro- or nanocapsules containing biocides, repellents, attractants, or plant growth regulators), revealed that around 3000 patents were registered worldwide in the last 10 years (2008–2018). Refining the search to consider stimuli-responsive materials reduced the number to about 200 published patents in the same period. The survey also showed that the number of patents concerning stimuli-responsive materials increased from 5 patents in 2008 to about 30 in 2018, with China and United States being the major countries from which applications were received.

**Fig. 2:**
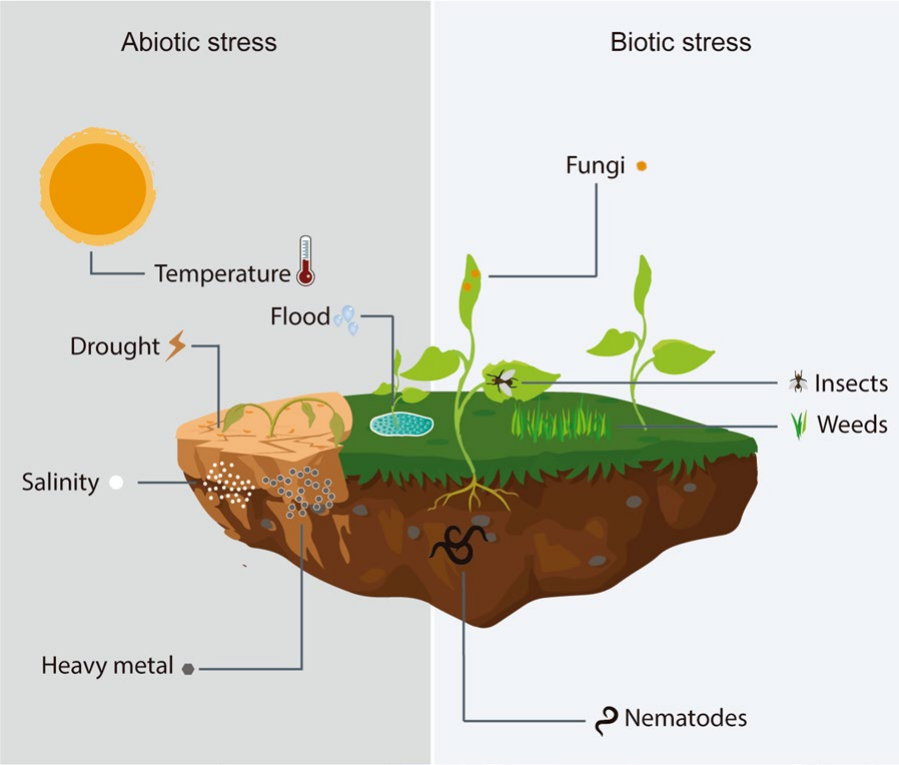
Abiotic and biotic factors enabling the use of stimuli-responsive nanomaterials for site-specific release of active substances, in order to increase plant resistance

The advantages of controlled release for the delivery of agrochemicals include reductions of phytotoxicity, leaching losses, volatilization, drift, and soil degradation, as well as increased safety during application [4]. However, the release of active agents from loaded particles mainly occurs by means of passive diffusion, capsule erosion, or osmotic pressure, resulting in poor control of pesticide release [3]. Hence, stimuli-responsive systems are promising candidates for improving controlled release properties, promoting site-specific and smart release of pesticides in response to biotic or abiotic stimuli [2] (Fig. 2). Biotic (plant pathogens, insects, and weeds) and abiotic (temperature, drought, flooding, and salinity) stressors are responsible for reducing global crop productivity by about 50% [5]. Therefore, prospecting new responsive carriers that respond to environmental stimuli such as pH [6–8], temperature [9, 10], redox conditions [11], light [12–14], and enzymes [3, 15] could be an important strategy for improving the resistance of crops to the stresses caused by these environmental factors.

The design of formulations using stimuli-sensitive carriers is a strategy to improve the controlled release system, where the macromolecules present an active response to small signals or modifications in the surrounding environment, resulting in changes in their physicochemical properties favoring the release of loaded substances [16, 17]. When used in agrochemical formulations, these sensitive carriers can allow the release of active compounds in response to abiotic or biotic stimuli such as light, pH, temperature, magnetic field, enzymes, among others [17, 18]. Stimuli-controlled delivery systems can be prepared using natural polymers (such as alginate, cyclodextrin, starch, chitosan, carboxymethylcellulose, and ethylcellulose) and silicas due to their wide availability and biodegradability [19]. Recently, carriers based on pillararenes and curcubit[n]urils were studied in pesticide and herbicide delivery due to their responsiveness to environmental stimuli [8, 20, 21].

Many physicochemical modifications can occur in the polymer structure, including changes in physical state, shape, conductivity, solubility, hydrophilic/lipophilic balance, and sol–gel transition. These changes can be reversible, such that when the stimulus is withdrawn, the polymer returns to its initial structure [16–18]. The release systems of different stimuli-responsive nanoparticles will be discussed in the next sections.

The use of stimuli-sensitive carriers in nanoformulations can enable the release of different compounds in an effective manner and at defined times, hence increasing bioefficacy, while reducing side effects, the dosage required, and the number of applications [16, 19, 22]. The use of stimuli-responsive particles is a promising technology that is still in an early stage of development and clearly needs further exploitation to extend its use in agroindustry. Therefore, the aim of this review is to discuss the current state of development of stimuli-responsive carrier systems, where the mechanisms of release of active ingredients are activated by different triggers, in response to biotic and abiotic factors, as well as to highlight the perspectives and commercial challenges for the use of smart delivery systems in the agricultural sector. In addition, this review indicates potential new opportunities and applications for stimuli-responsive carrier systems, contributing to the development of sustainable agricultural practices.

## Types of stimuli-responsive systems

### Systems responsive to pH and temperature

**Fig. 3:**
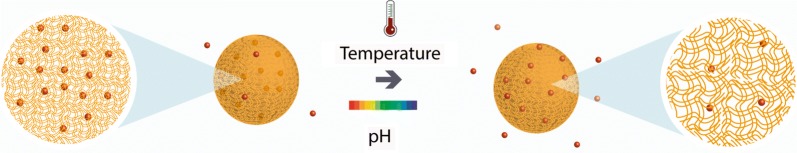
The release of substances promoted by modification on the carrier structure in response to pH and temperature changes

Nanocarrier release systems that respond to pH changes are based on the presence of ionizable functional groups in the carrier structure, such as pyrimidines, carboxylates, amines, phosphates, and sulfonates [23, 24]. At certain pH values, depending on the pKa of the carrier or the chemical molecule, the functional groups present a charge, which may be positive or negative, resulting in electrostatic interactions. The sizes of carriers sensitive to pH can be modified by the swelling or shrinking of the macromolecules in response to changes in the pH of the surrounding medium (Fig. 3). There are essentially two types of pH-sensitive materials: those having acidic groups in the structure (–COOH and −SO_3_H) and those having basic groups (–NH_2_), which swell at basic and acidic pH, respectively. Hence, the response can be the same, but the applied stimulus differs [25, 26].

Biopolymers are known for their properties such as biocompatibility, biodegradability, and nontoxicity, which are desirable for the development of nanocarrier systems used in eco-friendly formulations. Many of these biopolymers are ideal for the formation of nanocarriers that respond to pH stimulus [19, 27, 28].

The literature reports the development of several pH-responsive nanocarriers [19, 28–30]. For this type of system, a specific chemical molecule, such as a pesticide, can be encapsulated due to chemical (ionic or covalent) interactions, or be trapped in a polymeric mesh within the system during nanoparticle formation [31, 32]. A classic example of a pH-responsive nanocarrier system is based on alginate and chitosan [33, 34]. The alginate polymer chain contains carboxylic groups (pKa 4.8), so at basic pH the polymer is deprotonated [35] and possesses a negative charge. Chitosan is rich in amine groups (pKa 6.3), so at acidic pH it is protonated, with positively charged functional groups [36].

The ionic gelation method for the formation of nanocarriers composed of alginate and chitosan exploits the interaction between these two biopolymers. Solutions of the biopolymers are generally prepared at around pH 4.7, so that the carboxyl groups are attracted to the amine groups, forming cross-links between the polymers [31, 32]. The formation of this system is highly dependent on pH, which makes it pH-responsive. For example, pH changes, and consequently the protonation/deprotonation of the polymers, weaken or strengthen the electrostatic interactions, resulting in increase or decrease of the release of a chemical compound [29, 30, 37]. When a system is formed only by a single biopolymer, variation of the pH can result in repulsion between the polymer chains, due to the similar charges, leading to greater or lesser opening of the polymer mesh (swelling), hence affecting the release of the active agent [28]. These mechanisms summarize the way that most of the pH-responsive release systems function.

Other non-polymeric nanocarrier systems may be coated with pH-responsive biopolymers in order to prevent a release burst. Another strategy is functionalization of the surface of the nanoparticles by other compounds, linked by means of electrostatic interactions, in order to generate a response mechanism (gatekeeper) modulated by pH changes [6, 38, 39]. For example, silica nanoparticles present very rapid initial release of the incorporated ingredients, which could limit their applications. However, their surfaces are rich in hydroxyl groups, which permits the functionalization or coating of these carriers [23].

Generally, pH-responsive nanocarriers are also thermosensitive. Temperature-responsive nanocarrier systems have great potential for applications in agriculture, since the temperature constantly changes in the environment, directly affecting the emergence and development of agricultural pests such as insects, weeds, and fungi.

The release of active agents from temperature-responsive nanocarrier systems occurs due to temperature-dependent changes in the polymer physicochemical properties, allowing the release of the active ingredient and determination of the release kinetics [40, 41] (Fig. 3). These thermosensitive polymers are water-soluble at low temperatures but separate from the solution when the temperature exceeds the phase transition temperature, also known as the lower critical solution temperature (LCST). The main focus of studies of thermosensitive materials as drug delivery agents in controlled release systems is the phase transition temperature [41, 42].

High-viscosity water-soluble carriers are widely employed to achieve the controlled release of active ingredients. These include PEO-PPO-PEO block copolymers that are marketed as Poloxamers^®^ and Tetronics^®^ [43]. Poloxamers^®^ consist of a central PPO (polyoxypropylene) molecule, surrounded by two hydrophilic polyoxyethylene (PEO) chains. The structure of poloxamines (Tetronics^®^) is slightly different, with tetrafunctional block copolymers composed of four PEO-PPO blocks linked by a central ethylenediamine bridge [43, 44]. These polymers exhibit sol–gel phase transition below or near physiological body temperature [43, 45]. When dissolved in a liquid at low concentrations, they form monomolecular micelles, with increases in concentration leading to the formation of multimolecular aggregates. Above the critical gelation temperature, the micelles are arranged in a crystalline structure, hence giving rise to hydrogels [43, 45].

### Redox-responsive systems

**Fig. 4:**
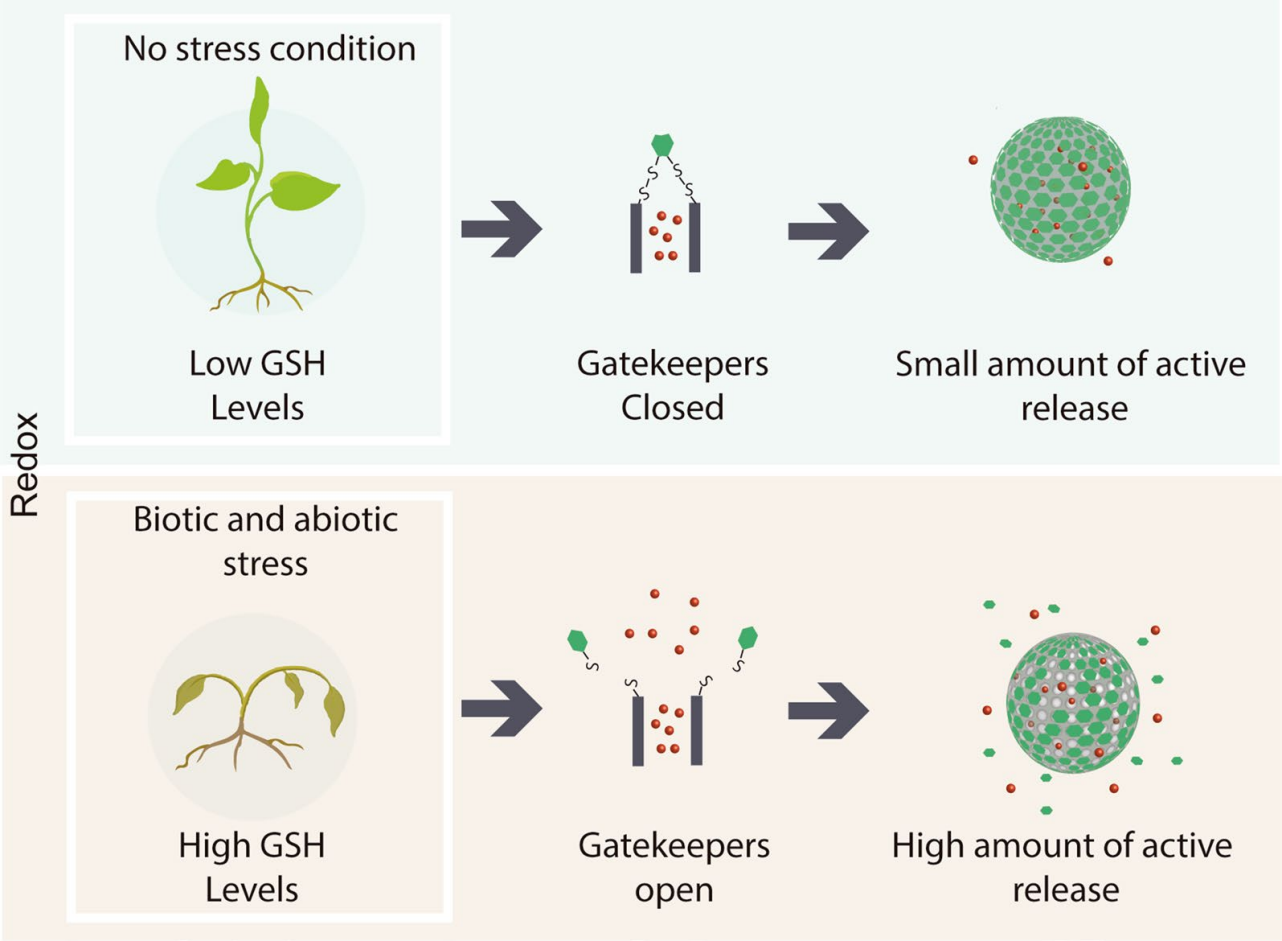
Release of active substances in response to redox stimuli. In normal environmental conditions the gatekeepers are linked, and the release is low, while under stress condition, the level of antioxidant increases and promote the cleavage of disulfide bonds, which enhance the release of loaded substances

In redox-responsive nanoparticle systems, the stimuli for release of the active compounds are endogenous factors present in the organism, such as antioxidant molecules, including glutathione [46]. Redox-responsive systems first emerged in the medical area, where they are used for the release of chemotherapeutic agents. Tumor cells have a higher concentration of reducing agents, compared to normal cells, and these molecules provide the trigger for the release. The release mechanism is based on the cleavage of a disulfide bond in the presence of the reducing agent. Therefore, it is necessary to functionalize the nanocarrier with thiol groups that are linked to the gatekeeper by means of disulfide bonds (Fig. 4). This is extremely important, since it is these links that will be cleaved [47].

Several redox-responsive systems have been developed, employing gatekeepers including organic components, inorganic crystals, polymers, and biomolecules [48]. Some examples of gatekeepers are cyclodextrins [49], curcumin [50], ZnO quantum dots [51], PEG [52], and hyaluronic acid [53].

### Enzyme-responsive systems

Enzyme-responsive materials have attracted considerable attention in the field of drug delivery, due to their high specificity and selectivity in response to internal biological stimuli [54]. However, studies using enzyme-responsive materials for the delivery of agrochemicals are still in the early stages. Enzymes play critical roles in all biological and metabolic processes, so exploiting enzymes as triggers for the smart delivery of agrochemicals has many advantages, such as specificity, accuracy, and efficiency, involving precise chemical reactions that occur under mild environmental conditions [2, 54].

Polymers that are sensitive to enzymes can interact with the biological surroundings, resulting in responses that can be detected by signal amplification, under certain conditions. The development of nanomaterials that can be stimulated by enzymes has attracted recent attention in several areas [54, 55]. The expression level of specific enzymes can be used as a trigger, resulting in an enzyme-mediated response of the nanomaterial and controlled release of active compounds in a site-specific manner.

**Fig. 5:**
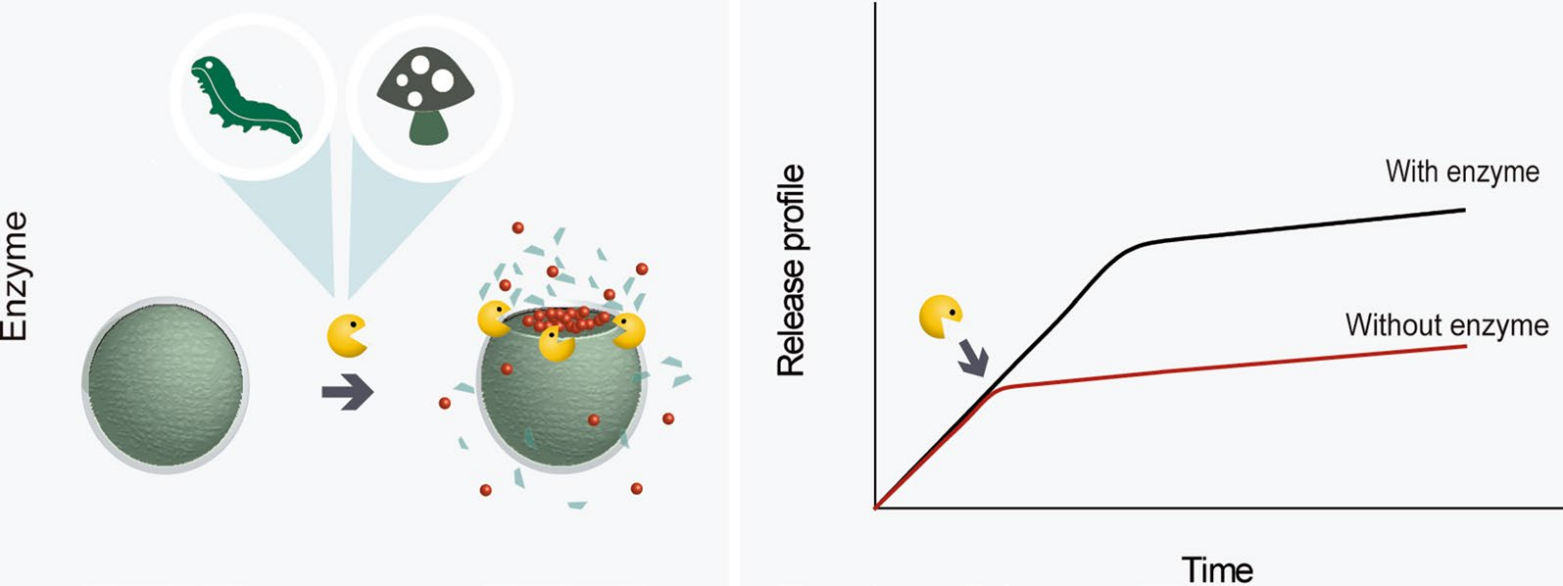
Release profile of active substances in response to the presence or absence of enzymes from insects or fungi

Many enzymes can be used as triggers for the release of active ingredients, in order to achieve efficient pest control. Special attention has been focused on enzymes present in the salivary glands and mid-gut of larvae and insects, in the soil, and produced by phytopathogenic fungi. The salivary glands and mid-gut of insects mainly contain carbohydrases and proteases [15, 56, 57]. In soil, the enzymes most frequently found are urease, alkaline phosphatase, dehydrogenase, and catalase [2]. Phytopathogenic fungi commonly release enzymes such as pectinases and cellulases, which are responsible for the degradation of plant cell walls [58]. The release system promoted by enzymes is described in Fig. 5.

### Photo-responsive systems

Photo-responsive nanoparticles allow spatiotemporal control of the release of active molecules by light irradiation. These nanoparticles can absorb light of different wavelengths in the UV, visible, and infrared ranges. Consequently, light-responsive nanoparticles have potential applications in the agricultural industry, since the abundance of sunlight radiation can trigger the release of active agents from the loaded particles [59].

The properties of polymer molecules that can be changed due to light stimulus include polarity, charge, conjugation, conformation, amphiphilicity, and optical chirality, among others. Such changes at the molecular level result in macroscopic changes in the properties of the polymers, such as shape, wettability, adhesion, optical properties, conductivity, solubility, and so on [40, 60]. The release of active compounds from these photo-responsive polymers, in response to light, begins instantaneously after structural modification of the nanocarrier by irradiation at a specific wavelength [60, 61].

**Fig. 6:**
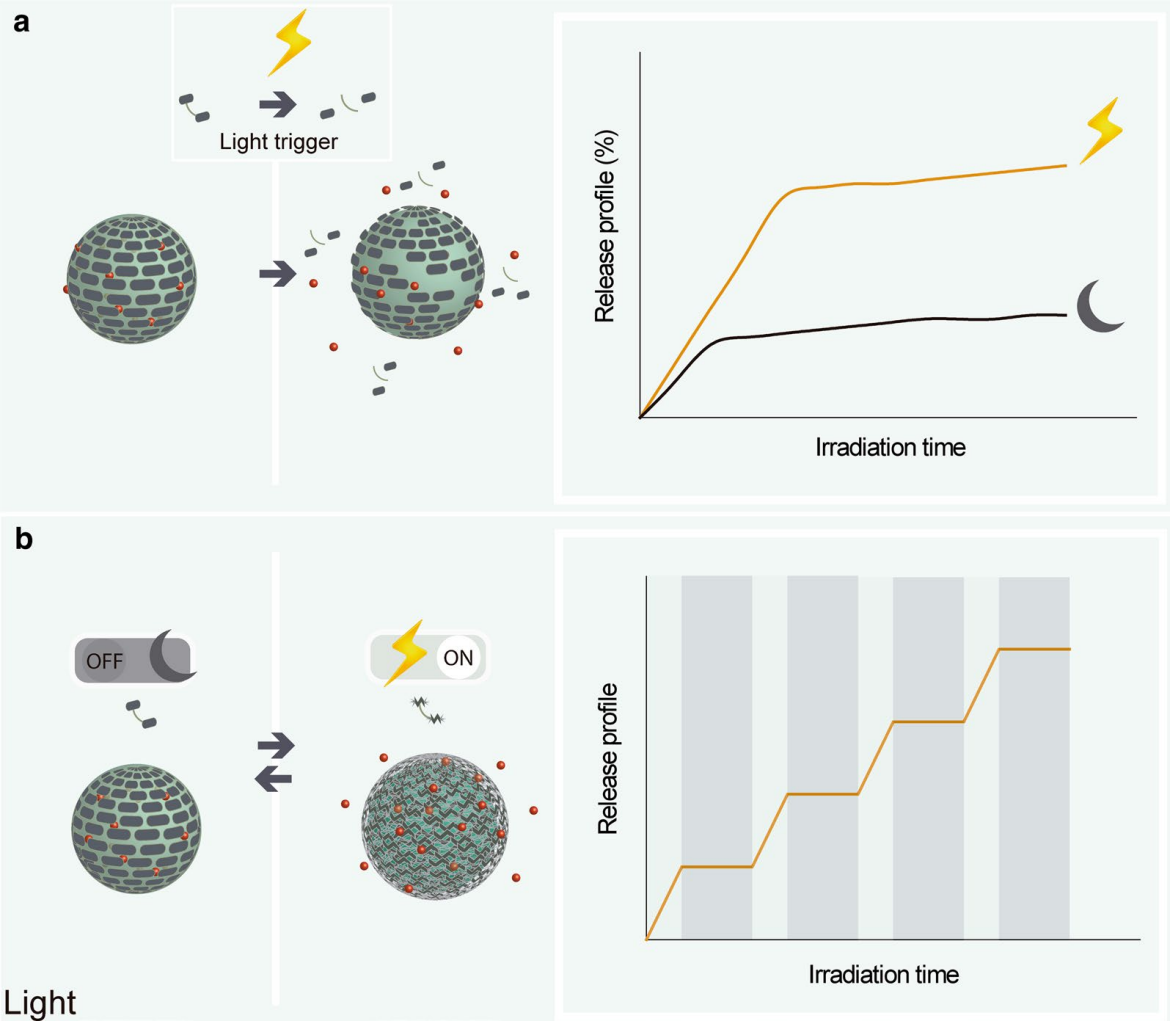
Light-controlled release of active substances in the release system stimulated by the presence of light irradiation (**a**); and in on/off system promoted by reversible changes in polymeric matrix (**b**)

Light-controlled systems can be obtained by the incorporation of photoactive molecules such as azobenzene, ortho-nitrobenzyl, coumarin, and spyropyrane in polymer-based materials. These molecules act as light-activated agitators that stimulate the release of the enclosed compounds, which can occur by either triggering degradation of the polymer or a linker between the polymer and a small molecule, or by changes in the polarity of the polymer. Coumarin and *o*-nitrobenzyl groups containing polymers can be cleaved into smaller molecules, while structures of azobenzene and spyropyrane polymers can be induced reversibly in the presence of certain wavelength light [62]. Light-controlled release can also act as an on/off system, where the presence of light stimulates the release of active compounds, while in the absence of light, or at a different light frequency, the system may be reversed and the release is interrupted [63] (Fig. 6).

## Applications of stimuli-responsive systems in agriculture

The development of stimuli-responsive smart delivery systems represents a new contribution to the goal of achieving sustainable agricultural practices. Stimuli-responsive nanoformulations can release their active ingredients in response to environmental triggers and biological demands more precisely, by means of targeted delivery or controlled release mechanisms. A variety of factors act to restrict crop growth and production, including biotic stress caused by insects, pathogenic diseases, nematodes, and weed competitors, as well as abiotic factors such as extreme temperatures, poor soils, unavailability or poor uptake of nutrients, metal-contaminated soils, drought, flooding, and high salinity [5, 64] (Fig. 2). Therefore, stimuli-responsive materials have been studied for the release of agrochemical compounds in response to pH, temperature, redox conditions, enzymes, and light. Table 1 summarizes the main advances in the development of stimuli-responsive systems for agricultural applications in order to overcome the adverse effects caused by biotic and abiotic stresses in plants.

**Table 1:** Stimuli-responsive nanoparticles for the delivery of agrochemicals in response to biotic and abiotic stressors

Material	Bioactive substances	Stimuli	Stimuli-responsive trigger	Conditions	Particle properties	Bioactivity	References
Silica	2,4-Dichlorophenoxy (herbicide)	pH/ionic strength/temperature	Trimethyl ammonium	–	423 nm 67.8 mV	Herbicidal activity in *Cucumis sativus* and reduction of leaching process	[76]
Chitosan and tripolyphosphate	Hexaconazole (fungicide)	pH	Chitosan	–	< 100 nm	Fungicidal activity against *R. solani* and cytotoxicity reduction	[28]
Silica	Triazolone (fungicide)	pH	Polydopamine and metal ions	–	1.5–10 nm	No biological activity assays	[93]
Lignin	Coumarin-6	pH	Lignin	–	100–400 nm	No biological activity assays	[6]
Chitosan	Copper ions (fungicide)	pH	Chitosan	–	361 nm 22 mV	Fungicidal activity against *C. luneta* and stimulation of the plant defense mechanism	[29]
Silica	Abamectin (insecticide)	pH	Polystyrene and-(trimethoxysilyl) propyl methacrylate	–	140 nm	Insecticidal activity against *Cnaphalocrocis medinalis* and increased leaf adhesion	[23]
Poly(succinate)	Nile red	pH	Functionalization with primary amines	–	8–83 nm	No biological activity assays	[27]
Zein	No active compounds	pH	Zein	–	210–297 nm	Soil degradation evaluation	[94]
Silica	Avermectin (insecticide)	pH and enzymes	Cyclodextrin	–	380–400 nm	Insecticidal activity against *Plutella xylostella*	[15]
Alginate and chitosan	Acetamiprid (insecticide)	pH	Alginate and chitosan	–	201 nm − 32 mV	No biological activity assays	[66]
Silica	Gibberellic acid (plant growth regulator)	pH/metabolites and ultrasound	Iron nanoparticles	–	139–189 nm	Increase in germination rate of cabbage and *Arabidopsis thaliana*	[8]
ɣ-Polyglutamic acid and chitosan	Avermectin (nematicide)	pH	ɣ-Polyglutamic acid and chitosan	–	56–62 nm	Nematicidal activity	[30]
Silica	Prochloraz (fungicide)	pH/temperature/enzymes	Chitosan	–	340 nm 34 mV	Fungicidal activity and reduction of toxicity to zebrafish	[39]
Alginate	Cypermethrin (fungicide)	pH	Alginate	–	115–119 nm − 21 mV	Reductions of leaching and phytotoxicity	[35]
Silica	Diquat (herbicide)	pH	Functionalization with sulfonated groups	–	240 nm − 17 mV	Herbicidal activity against *Datura stramonium* L.	[95]
Graphene oxide	Salicylaldehyde	pH	Functionalization with hydrazine	–	300 nm	No biological activity assays	[73]
Graphene oxide	Imidazole (fungicide)	pH	Polydopamine	–	− 30 mV	Fungicidal activity against *Fusarium oxysporum* f. sp. *cucumerinum* and reduction of leaching process	[71]
Poly(succinate) and glycine	Avermectin (insecticide/nematicide)	pH	Glycine methyl ester	–	56 nm	Activity against *Plutella xylostella* and high leaf adhesion	[65]
Silica	Curcumin	pH	Chitosan	–	139–222 nm	Antimicrobial activity against *Staphylococcus aureus* and *Escherichia coli*	[36]
Polydopamine and attapulgite	Chlorpyrifos (insecticide)	pH	Alginate	–	20 nm	Increased larval mortality	[96]
Silica	Azoxystrobin (fungicide)	pH	Chitosan	–	152 nm	Fungicidal activity against *Phytophthora infestans*	[37]
Silica	Abscisic acid (plant growth regulator)	Redox	Disulfide bond with decanethiol	Increase of glutathione concentration	20 nm	Plant development, reduction of hydric stress, and induction of sustained expression of defense gene (AtGALK2) in *Arabidopsis*	[48]
Silica	Salicylic acid (plant growth regulator)	Redox	Disulfide bond with decanethiol	Increase of glutathione concentration	85 nm	Induction of sustained expression of defense gene (PR-1) in *Arabidopsis*	[46]
Chitosan	Gibberellic acid (plant growth regulator)	Temperature	Alginate/chitosan	–	195–450 nm	Increased seed germination and plant development	[79]
Silica	Thymol (fungicide)	pH/temperature	Carboxylic, amino, and hydroxyl groups	–	200 nm	No biological activity assays	[74]
Attapulgite nanocomposite	Herbicide	Temperature	Glyphosate	–	0.5–1000 nm	Herbicide activity/high leaf adhesion	[9]
Polymeric micelle	Insecticide	Temperature	Pyrethrin	–	80–130 nm	Higher larvicidal activity against *Culex pipiens pallens*	[67]
Biochar and attapulgite	Herbicide	Light	Azobenzene	UV, Vis, UV–Vis, and sunlight	–	93.7% of Bermuda weed was controlled with herbicide particles under UV–Vis exposure	[13]
Polyethylene glycol	Herbicide		3-Nitro-4-bromomethylbenzoic acid	UV light (365 nm)	51 to ~ 63 nm	–	[77]
Carboxymethyl chitosan	Herbicide		2-nitrobenzyl succinate	UV light (365 nm) and sunlight	196 nm 26.2 mV EE: 91.9%	–	[78]
Photo-removable protecting group	Herbicide		Coumarin	UV–Vis light (310, 350, and 410 nm)	–	*Vigna radiata* growth inhibition	[69]
	Plant growth regulator		Coumarin	Sunlight	–	Promoted shoot and root growth of *Cicer arietinum*	[80]
	Sex pheromone		Coumarin, pyrene, anthracene	UV and sunlight	–	Promoted better attraction of moths (*Chilo partellus*), compared to the free compounds	[70]
	Insecticide		Coumarin	Blue light (420 nm) or sunlight	–	Insecticidal effects against *Aphis craccivora* under light exposure	[68]
	Insecticide		Coumarin	Blue light (420 nm) or sunlight	–	Insecticidal effects against *Mythimna separata* under blue light exposure	[12]
Acrylate and polyethylene glycol	Herbicide		Coumarin	UV light (310 nm)	–	Inhibition of root growth of *Curcubita maxima*	[14]
Cucurbut[8]uril	Herbicide		Azobenzene	Sun light (360–800 nm)	187 nm − 21.7 mV EE: 16.4%	Paraquat-loaded vesicles were efficient in controlling *Estuca arundinaceae* with a quick release of herbicide	[21]
Polydopamine capped with PNIPAm	Insecticide	Near-infrared laser/temperature	Photothermal polydopamine	NIR irradiation (808 nm, at 2 W/cm^2^) and temperature at 40 °C	~ 250 nm	–	[97]
Graphene oxide coated with polydopamine	Fungicide	Near-infrared laser/pH	–	NIR laser (808 nm, 1.5 W/cm^2^), pH 9	− 30.5 mV	Activity against *Fusarium oxysporum*	[71]
Silica and carboxymethylcellulose	Insecticide	Enzyme	Cellulase	pH 7 at 25 °C	1 to ~ 3 μm EE: 35%	Cellulase-responsive properties with sustainable insecticidal activity against *Myzus persicae*	[3]
Silica and pectin	Antibiotic		Pectinase and glutathione	pH 7 at 25 °C	1 to ~ 2 μm EE: 20%	Improved efficacy of kasugamycin against *Erwinia carotovora*	[58]
Hollow mesoporous silica and cyclodextrin	Insecticide		α-Amylase	pH 7 at 25 °C	400 nm − 36 mV EE: 38%	Enhanced the stability and activity of avermectin against *Plutella xylostella*	[15]
Isocyanate-functionalized mesoporous silica cross-linked with polyethylenimine	Herbicide		Urease	pH 7 at 25 °C	3 to ~ 5 μm EE: 30%	The microcapsules increased herbicidal duration and activity against *Echinochloa crus*-*galli* and *Amaranthus retroflexus*	[2]

*EE* Encapsulation efficiency

### Examples of stimuli-responsive nanocarriers for insect and nematode control

#### pH-responsive systems

Systems that are pH-responsive can be developed for the control of major agricultural pests such as insects and nematodes, since their intestines present a range of acid and alkaline pH conditions that enable activation of the release mechanism after ingestion of the nanocarriers [15, 65]. For example, Patel et al. [35] developed an alginate nanocarrier system for the insecticide cypermethrin. At acidic pH, the high degree of ionization of the alginate polymer led to greater interaction with calcium ions, increasing the cross-linking of the polymer mesh, hence making the interior of the nanoparticles more hydrophobic and reducing release of the insecticide. When the pH increased, the electrostatic interactions diminished, allowing the entry of water into the system and consequent release of the active agent.

Other pH-responsive systems have been developed for insecticides. In the study of Gao et al. [23], a system of silica nanoparticles coated and functionalized with polystyrene and (trimethoxysilyl)propyl methacrylate was developed for delivery of the insecticide abamectin. The results showed that after 15 days at pH 5 and 7, there was 14–15% release, demonstrating that the system prevented premature release of the insecticide, whereas at pH 10, release of the insecticide reached 39% after 5 h and 87% after 15 days.

The system developed by Kaziem et al. [15] showed the same features for cyclodextrin-silica nanoparticles containing avermectin. Although the system was stimulated by enzymes (α-amylase), it also presented pH-responsive behavior. At alkaline pH, there was 40% release after 17 days, while release of around 6% was obtained at neutral and acid pH, over the same period. Another system developed for the pH-responsive release of avermectin was proposed by Wang et al. [65], where a poly(succinate) system containing nanoparticles showed release percentages of 34, 62, and 85% at pH 5.5, 7, and 8.5, respectively. In this case, the response was not due to electrostatic interaction effects, but because increase in pH led to collapse of the nanoparticles, consequently releasing the insecticide.

Kumar et al. [66] described an alginate and chitosan system containing the insecticide acetamiprid, where 50% of the insecticide was released after 24 h at pH 10, while the same amount was released after 36 h at pH 7 and 4. In the same study, the authors evaluated the release of the insecticide in alkaline soils (pH 8.3), which showed the same release profile as in pH 10 buffer.

Similarly, pH-responsive systems can be used for other pests that damage agricultural crops, such as nematodes. For example, Liang et al. [30], developed a nanoparticle system consisting of γ-polyglutamic acid and chitosan for the transport of avermectin, used as a nematicide. The system showed an initial release of 20%, independent of pH, while subsequently there was release of 69.5% at pH 8.5, compared to 60.4% at pH 7 and 57.5% at pH 5.5. The nanocarrier system employed showed high stability at pH 5.5, due to the electrostatic interactions between chitosan and the carboxyl groups of γ-polyglutamic acid. As the pH increased, this interaction diminished, hence increasing the release of the nematicide.

In agricultural systems, pH variations occur in soils, in different plant organs, during physiological processes of the plant, and in the maturation of fruits, as well as due to the presence of pathogens or agricultural pests [23, 29]. Therefore, nanocarrier systems able to release active agents in response to pH changes represent a major strategy for the development of new nanopesticides, since the release kinetics is modulated according to pH changes.

#### Temperature-responsive systems

Zhang et al. [67] developed a release system for the insecticide pyrethrin, employing temperature-responsive nanocarriers consisting of nanomycetes. The release was evaluated using three temperature conditions (13, 18, and 26 °C), simulating the temperatures of the mosquito developmental stages in water. At low temperature, the system released 12% of the pyrethrin in the first 16 h, with no further changes after this period. At higher temperatures, the release increased over time, reaching 31.9% at 18 °C and 49.7% at 26 °C.

Liang et al. [39] coated silica nanoparticles with temperature-responsive chitosan and showed that higher release of avermectin was obtained by raising the temperature, with release of 18.82% at 25 °C and 34.21% at 50 °C, demonstrating that coating with chitosan was a good option for altering the release profile according to temperature. The greater release at higher temperature was due to changes in the thermodynamic movement of the molecules, which increased diffusion of the pesticide through the chitosan.

#### Enzyme-responsive systems

Enzyme-responsive polymers have been studied for the release of insecticides in the presence of enzymes found in the guts of insects and larvae. Guo et al. [3] reported the synthesis of microcapsules containing emmamectin benzoate, using silica cross-linked with epichlorohydrin-modified carboxymethylcellulose. The release behavior of the microcapsules was evaluated in the presence of cellulase, which cleaved the cellulose wall material into smaller fragments, releasing the active ingredient. Almost 30% of the emmamectin was released after 1 h of cellulase exposure, with around 80% release reached after 30 h. In the absence of enzymes, less than 20% was released after 30 h. Use of the silica-epichlorohydrin-carboxymethylcellulose microcapsules reduced the genotoxic effects of the active agent and enhanced the insecticidal activity against *Myzus persicae*, compared to the use of emmamectin benzoate alone.

Kaziem et al. [15] investigated the use of an α-amylase-responsive carrier for the development of a smart delivery system that responded to internal stimuli produced by insects, triggering biocide release. The system developed was based on α-cyclodextrin anchored on hollow mesoporous silica loaded with avermectin. The nanoparticles showed a strong response to the presence of α-amylase, with avermectin release of 41.64% up to day 17, compared with only 5.88% release in the absence of α-amylase, during the same period. The mortality of larvae remained higher than 80%, even after 14 days, while the effectiveness of a commercial avermectin formulation decreased to 43.4%. The avermectin nanoformulation remained active 44 days after application, when 6.63% larvae mortality was observed.

#### Photo-responsive systems

Coumarin was investigated as a photo-trigger for insecticide delivery by Gao et al. [12] and Xu et al. [68]. Advantageous properties of coumarin include its ability to improve the stability of molecules, together with pesticidal activity, strong fluorescence, fast release rates, broad absorption wavelength range, and high biocompatibility [12, 69]. Gao et al. [12] described, for the first time, a photocaged insecticide composed of coumarin and fipronil. A covalent link was established between fipronil and coumarin, resulting in an on/off system where the insecticide release was light dependent. The system was able to provide efficient control of *Aedes* larvae, when exposed to blue light (LC_50_ = 0.56 μmol L^−1^) and sunlight (LC_50_ = 0.37 μmol L^−1^). In similar work, Xu et al. [68] obtained a caged insecticide using spirotetramat-enol and coumarin, with covalent linking. The photo-triggered release profile was evaluated by exposing the system to blue light (420 nm) and sunlight, which showed efficient release of spirotetramat-enol using both irradiation sources. In the dark, the caged insecticide showed low insecticidal activity (LC_50_ = 1 mmol L^−1^) against *Aphis craccivora*, while blue light irradiation significantly increased mortality (LC_50_ = 0.11 mmol L^−1^).

In other work [70], a sex pheromone used as an insect attractant was caged using different photo-removable protecting groups, including 7-hydroxy-4-hydroxymethylcoumarin, 1-pyrenemethanol, 9-anthracenemethanol, and 2-(hydroxymethyl) anthraquinone. All the photoactive molecules were able to release the pheromone, with UV (≥ 350 nm) or sunlight irradiation being essential for the controlled release of (Z)-11-hexadecen-1-ol.

Tong and coworkers [71] proposed the use of infrared stimuli-responsive nanocomposites to prevent losses of hydrophilic pesticides and improve their efficiency. For this, graphene oxide (GO) was loaded with hymexazol (Hy) by absorption, followed by coating the GO surface with polydopamine (PDA). The Hy-GO@PDA nanocomposite presented a response to photothermal heating using a near-infrared (NIR) laser at 808 nm. The release behavior showed that the Hy-GO@PDA was responsive to both pH and NIR, so the release of Hy could be controlled by either pH or NIR irradiation. The highest Hy release was 75% after 120 h, at pH 9.0, under NIR irradiation.

Photo-responsive formulations have broad potential applications in agroindustry. In this emerging field, the development of new cost-effective sunlight-triggered delivery devices is highly desirable for the encapsulation of fertilizers, pesticides, and plant growth regulators.

### Examples of stimuli-responsive nanocarriers for control of phytopathogens

#### pH-responsive systems

Systems that are pH-responsive are also being studied for the control of diseases caused by phytopathogens. An example is a system based on poly(succinate), which is a biodegradable hydrophilic biopolymer that is liable to hydrolysis at alkaline pH [36]. Hill et al. [27, 72] developed a poly(succinate) nanocarrier system intended for the control of diseases affecting plant phloem. The pH of most plant tissues is acidic, while that of the vascular phloem tissue is alkaline. Therefore, this type of nanocarrier system could be used against pathogens such as *Citrus huanglongbing* (known as greening), which specifically infects the phloem, leading to death of the plant.

Systems responsive to pH could also be used to prevent the contamination of fruits by pathogens, which can reduce yields by up to 30%. Contamination of fruits by fungi results in lowering of pH, due to the production of enzymes such as esterases and pectinases [39]. Fruit ripening may also be accompanied by increasingly acidic pH [73]. Nanocarrier systems can be developed to control the growth of contaminant organisms in food, as well as to avoid early maturation. For example, Liang et al. [39] developed silica nanoparticles coated with chitosan for delivery of the fungicide prochloraz. In the absence of the esterase enzyme and at pH 7, only 18.9% of the fungicide was released for 30 days. However, decrease of the pH and the presence of the enzyme stimulated the system, with 82% release obtained during the same period. At acidic pH, chitosan becomes protonated, exposing the pores of the silica nanoparticles and consequently enabling release of the active agent. Sharma et al. [73] described a system of graphene oxide nanoparticles for the release of salicylaldehyde, where the binding of salicylaldehyde to the graphene oxide nanoparticles was mediated by the hydrazone molecule. At acidic pH, 50% of the active agent was released after 50 h, compared to only 15% at neutral pH. The release was due to the hydrolysis of hydrazone at acidic pH, releasing the active agent and reducing precocious ripening.

Wu et al. [36] incorporated silica nanoparticles containing curcumin in chitosan gel, in order to produce an intelligent food packaging system. The use of such systems with food can prevent the growth of harmful organisms. The system showed faster release under acidic conditions (pH 2), compared to neutral conditions (pH 6 and 7.4). At acidic pH, chitosan is protonated, resulting in electrostatic repulsion between the polymer chains, hence increasing the volume of the polymer mesh and releasing the active agent, in contrast to neutral pH, where chitosan is in a neutral state.

#### Redox-responsive systems

Yi et al. [46] developed a redox-responsive system for the plant growth regulator salicylic acid, which is a hormone that regulates plant defense mechanisms in the presence of biotrophic pathogens. Silica nanoparticles were functionalized with thiol groups, followed by coating with decanediol, forming specific disulfide bonds that could be cleaved in the presence of glutathione. In in vitro release assays, there was an increase in glutathione levels, which increased the release of salicylic acid. Plant assays, using *Arabidopsis* as a biological model, evaluated expression of the PR-1 defense gene that is mediated by salicylic acid. Increase of the glutathione concentration in the plants resulted in the nanocarrier system starting to release the salicylic acid, which induced high expression of the PR-1 gene that can increase the resistance of plants against pests.

#### Temperature-responsive systems

Mattos et al. [74] developed silica nanoparticles containing the fungicide thymol, with functionalization using –OH, –NH_2_, and –COOH groups. When the temperature was increased, the nanoparticles functionalized with amino groups showed higher release of the fungicide, compared to the nanoparticles functionalized with hydroxyl and carboxyl groups.

#### Enzyme-responsive systems

Pectinases from phytopathogenic fungi have been investigated for triggering the release of antibiotics in order to control plant diseases. Liu et al. [75] synthesized kasugamycin conjugated with modified pectin by amide bonds and evaluated its triggered release performance in the presence of *Pseudomonas syringae* pv. *lachrymans*. A sustained-release profile was observed for kasugamycin-pectin, which was controlled by the pectin degradation enzymes produced by *P. syringae*. The release rate of kasugamycin increased with time, indicating that greater quantities of pectinase were released during microbial growth, resulting in faster release of the active compound.

In other work, Liu et al. [58] described the preparation of enzyme-responsive kasugamycin microcapsules, using mesoporous silica conjugated with pectin by addition of disulfide bonds. Dual-responsive kasugamycin microcapsules were obtained, with protective effects against degradation by temperature and light. The delivery system was triggered by either pectinase or gluthatione stimuli, with the release of kasugamycin reaching 88.13% after 30 h in the presence of both stimulants, while values of 68.87% and 77.74% were obtained in the presence of pectinase or gluthatione alone, respectively. The antimicrobial efficacy of the kasugamycin microcapsules against *Erwinia carotovora* was lower during the first few days, subsequently increasing over time due to continuing production of the triggers by the microorganism. It was concluded that the product could remain effective during approximately 3 weeks, indicating its great potential for use in agricultural applications.

### Examples of stimuli-responsive nanocarriers for weed control

#### Temperature-responsive systems

A temperature-responsive nanocarrier for the herbicide glyphosate was proposed by Chi et al. [9], based on a nanocomposite composed of attapulgite, NH_4_HCO_3_, amino silicon oil, and poly(vinyl alcohol). Increase of the temperature resulted in the formation of pores in the nanocomposite, due to dissolution of the poly(vinyl alcohol) surfactant, facilitating release of the herbicide. After 72 h, the amounts released were 13% at 25 °C, 22% at 40 °C, and 33% at 50 °C.

In the case of silica nanoparticles, the temperature affects the diffusion or thermodynamic movement of the guest compound through the silica pores. Strategies such as functionalization with different functional groups, or coating with polymers, can be used to modulate the release from the nanoparticles at different temperatures. For example, Cao et al. [76] reported that release of the herbicide 2,4-dichlorophenoxy from silica nanoparticles increased at higher temperatures, due to greater diffusion of the herbicide through the nanoparticle pores to the external medium. The amounts of the active agent released after 900 min were 96, 75, and 52% at temperatures of 40, 30, and 20 °C, respectively.

#### Enzyme-responsive systems

Soil enzymes can also be useful tools for triggering the release of bioactive compounds such as pesticides and fertilizers. For example, urease was found to be an effective trigger for the release of herbicide from silica nanoparticles [2]. Urease-responsive herbicide microcapsules were obtained by conjugating pendimethalin (PEI) onto the surface of isocyanate-functionalized mesoporous silica by means of urea bonds, which provided sites for cleavage by urease. This system, denoted silica-IPTS-PEI, showed cumulative release of 81.94% after 30 h, while release of only around 10% was achieved in the absence of urease. In addition, the formulation improved the durability of PEI applied for weed control.

Therefore, enzyme-responsive formulations offer considerable benefits in agriculture. The active compounds will be mostly released only in the presence of enzymes, hence increasing the efficiency and safety of the product, while reducing the effects on nontarget organisms. An additional important consideration is that the use of such responsive formulations could substantially reduce the amounts of chemicals applied, compared to conventional techniques.

#### Photo-responsive systems

Recent work [13] reported the development of a light-responsive controlled-release system, where particles produced using biochar, attapulgite, azobenzene, and amino silicon were used to encapsulate the herbicide glyphosate. Azobenzene was used as a light-stimulated trigger, since it has *trans* and *cis* isomers that can be transformed to each other under UV or visible light, consequently promoting release of the herbicide. Continuous release of glyphosate was obtained, which was more strongly induced by UV–Vis irradiation (around 100% glyphosate release), compared to exposure to daylight or dark conditions (around 80% release). This effect was attributed to the simultaneous *trans*–*cis* and *cis*–*trans* transformations of the azobenzene molecules, induced by UV–Vis irradiation, which did not occur in daylight or in the dark. The particles also showed high leaf surface adhesion, due to the high viscosity of amino silicon, as well as stability under different pH conditions. Weed control efficiencies of 93.7 and 46.7% were achieved when the light-responsive particles were used in the presence and absence of UV–Vis light, respectively, confirming the stimulus provided by azobenzene in the presence of irradiation.

Gao and co-workers [21] proposed a photo-responsive supramolecular vesicle loaded with paraquat. The vesicle was prepared by the self-assembly between cucurbit [8] uril, paraquat, and an azobenzene derivative. The vesicles showed good stability properties when storage in dark for 210 days, with a slight increase in particles diameter from 208.1 to 293.5 nm. The cumulative release ratio showed the UV responsiveness of paraquat-loaded vesicles, where in the dark only about 10% of paraquat was released after 10 h, and when exposed to UV light, the release of paraquat reached 90% after 24 min, under continuous irradiation. The authors also showed that the loaded herbicide showed safer profile than free paraquat in cell and animal models, and similar herbicidal activity than compared with free herbicide against *Estuca arundinaceae*.

The photoactive molecule 3-nitro-4-bromomethylbenzoic acid (NBA) was used as a photo-trigger in the synthesis of a system consisting of polyethylene glycol (PEG) and the pesticide dichlorphenoxyacetic acid (2,4-d). The 2,4-d-NBA-PEG system could self-assemble into core–shell micelles, for which the release profile showed the controlled release of 2,4-d under exposure to sunlight (99.6% in 8 h) or UV irradiation (99.5% in 9 min), while no release was observed in the absence of irradiation [77]. Ye et al. [78] successfully used the photolabile 2-nitrobenzyl group conjugated with carboxymethyl chitosan to synthesize photo-responsive micelles for delivery and controlled release of the hydrophobic herbicide diuron. The cross-linked micelles released the herbicide in response to the stimuli of UV irradiation and sunlight, indicating their potential as a promising tool for the delivery of pest inhibitors.

Other work has investigated the caging of 2,4-d with coumarin [69], as well as the synthesis of a coumarin copolymer based on acrylate and polyethylene glycol loaded with 2,4-d, which presented advantages including stability, release of 2,4-d controlled by UV light, and extended herbicidal activity [14].

### Examples of stimuli-responsive nanocarriers for soil applications

The quality, nutritional level, and physicochemical properties of soil play important roles in the growth and development of plants [64]. Stimuli-responsive nanoparticles can be applied to soils in order to provide targeted and slow release of fertilizers, fungicides, herbicides, and plant growth regulators. Sun et al. [48] developed a redox-responsive system for encapsulation of abscisic acid, a plant growth regulator responsible for regulating physiological changes in plants and increasing glutathione levels under stress conditions. Trials using *Arabidopsis* plants under hydric stress conditions showed that treatment with the nanoparticles induced physiological changes, with closure of the stomata in order to avoid water loss, as well as high expression of the AtGALK2 gene related to stress relief. The plants treated with nanoparticles presented a higher survival rate under hydric stress.

Li et al. [8] recently developed an interesting multi-responsive nanoparticle system for the delivery of gibberellic acid (GA_3_). The exogenous application of this plant growth regulator can alleviate plant stress caused by drought and salinity. GA_3_ was enclosed in hollow mesoporous silica (HMS) capped with Fe_3_O_4_ nanoparticles functionalized with carboxylatopillar[5]arene ammonium (WP[5]A). The release system could be controlled by various stimuli, including alkaline or acid pH, polyamines, and ultrasound. The stimulus provided by polyamine was due to competition between 1,4-butanediamine and pyridine for interaction with WP[5]A. The ultrasound stimulus was due to the weakening and destruction of host–guest interactions, which could be restored by removing the ultrasound source.

Chitosan biopolymers responsive to temperature changes have also been evaluated for plant growth regulator release [79]. It was shown that the temperature could alter the viscosity and hydrophobicity of the polymers, hence modulating the release of GA_3_, with faster release when the temperature was increased to 30 °C, compared to the release at 25 °C.

In another model, a photo-responsive system was developed using the photo-removable protecting group of coumarin for the smart release of plant growth regulators such as indoleacetic acid and naphthoxyacetic acid. The system showed controlled release of the plant growth promoters when exposed to sunlight, which resulted in improved root and shoot growth of *Cicer arietinum* [80].

A novel stimuli-responsive nanoparticle was developed for controlled fertilizer release in response to anion exchange. Given the high availability of ions in the soil, Zhang et al. [81] developed an anion-responsive nanosystem based on polyethylenimine-modified hollow mesoporous silica. The nanosystem was obtained by electrostatic interaction between the carrier and the fertilizer (selenium). The release of selenate was significantly stimulated by valence and the anion content, with maximum release of selenate (90% after 10 h) obtained using 5 mM phosphate solution. This condition also improved plant growth by 60.7%, compared to the control, indicating that selenate was successfully transformed into organic Se by the plant, with enhanced efficiency and avoidance of losses.

Systems employing magnetic stimuli represent another promising technology that could be used for the delivery of hormones and biocides by remote control. A magnetic nanosystem has been reported for drug delivery [82], while for agricultural purposes, studies of magnetic nanoparticles have so far focused on soil decontamination [83, 84].

## Commercial limitations and challenges

**Fig. 7:**
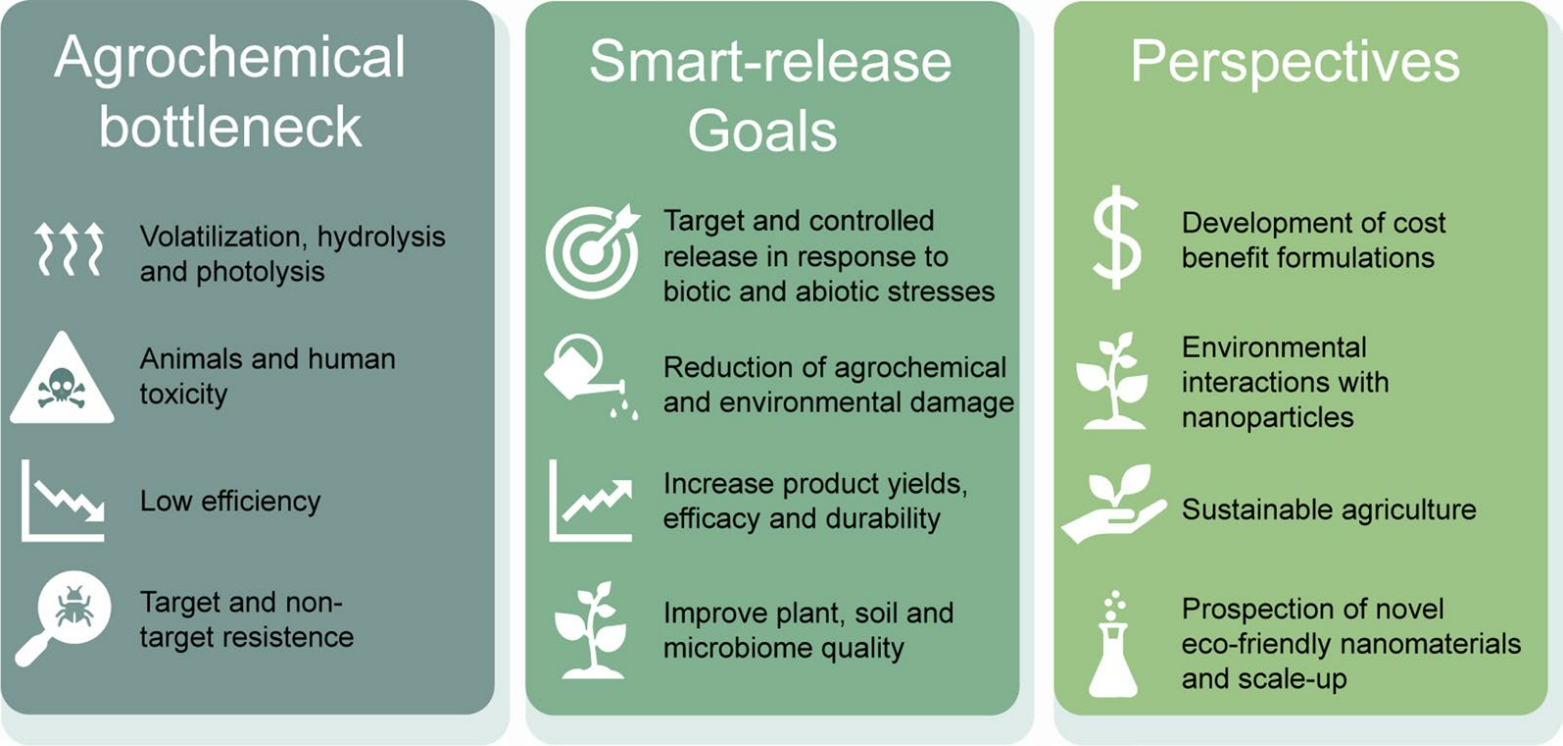
Advantages of stimuli-responsive nanoformulations, compared to conventional agrochemicals, and perspectives for improving their use in agriculture

Many publications have shown that nano-based pesticides have great potential in the field of pest management, offering advantages for the environment and human health, compared to the use of conventional pesticides [85]. Figure 7 summarizes the main problems associated with conventional pesticides, indicating the ways that stimuli-responsive release nanoformulations could help to overcome them. Figure 7 also shows some key areas where stimuli-responsive systems need to be improved in order to be successfully marketed.

Although nanotechnology can bring many advantages to the agricultural sector, there are few nanotechnology-based products available on the market [86, 87]. One of the factors influencing this low level of commercialization is that the great majority of studies are undertaken in universities and research institutes, or by small companies (spin offs and start-ups) set up for specific agricultural purposes. At the same time, large companies possess many patents, with the number increasing annually. New nano-based products for the agricultural sector do not reach the market, because the large companies are accumulating patents and waiting for opportunities for future exploitation, following the development of promising commercial products [88, 89].

Another issue concern understanding the risks related to the use of nano-based pesticides, which are complex and poorly understood. It is already known that the transport, bioaccumulation, and rate of degradation of nano-based pesticides are different, compared to conventional pesticides, but further work is needed to elucidate these issues. There are limited data available concerning the effects of nano-based pesticides on crop health, soil biodiversity, nontarget organisms, and human health [86, 87]. Furthermore, the development of nano-based pesticides suffers from a lack of effective strategies to understand their mechanisms of action. It is very important to evaluate the fate of nano-based pesticides in the environment, in order to elucidate the interactions of these materials with terrestrial and aquatic organisms [90].

In addition to the problems mentioned above, it should be highlighted that economic issues remain a major hindrance for the commercialization of nanopesticides [91]. The initial costs involved in developing nanopesticides are high, with positive financial returns only being possible if large quantities of these products were to be used in crop protection, which is not the currently the case. In addition, the lack of regulation is a major impediment to the expansion of nanotechnology in agriculture. Another factor that limits the commercialization of nano-based pesticides is the high cost associated with registering a new active compound [88, 92].

The most significant challenges that need to be overcome in order to increase the commercialization of nano-based pesticides can be listed as follows: (a) development of standardized methods for reliable assessment of risk–benefit; (b) advances in knowledge of the interactions between nanomaterials and plants or pathogens; (c) development of strategies to track nanopesticides in the environment and evaluate their impacts on food security and human health; (d) lack of a clear and standardized definition of nanomaterials; (e) development and implementation of legislation at an international level to ensure the safe development and application of stimuli-responsive nanopesticides; and (f) lack of a global network for effective communication among the public and private organizations engaged in the development of nano-based products [5, 85–87]. It is clear that further in-depth research will be required in order to address these issues, prior to full commercialization of nano-based products. It is especially important that there should be interaction among all the potential stakeholders, including universities, research institutes, companies, governments, NGOs, and consumers.

## Conclusions and perspectives

The application of nanotechnology in agriculture has grown exponentially in the last few years. Many commercial companies are now forming partnerships with scientific communities in order to develop feasible nanopesticides. A hotspot of agricultural nanotechnology is the development of stimuli-responsive nanoformulations able to maintain the stability of the active ingredient under environmental conditions, deliver the active ingredient to the target, decrease its dispersal in the environment, and prolong its biological activity. Currently, although stimuli-responsive systems are well developed in the area of medicine, their applications in agriculture remain limited. The pesticide field also requires continued systematic research for the development of improved environmentally responsive, targeted, controlled-release pesticide formulations. Effective systems that are responsive to internal bio-stimulation present a great challenge in this respect. Although stimuli-responsive characteristics could reduce the premature degradation of pesticides, improve their efficacy, and decrease collateral effects towards nontarget organisms, there are many drawbacks that hinder their large-scale applications, as discussed throughout this review. In order to ensure the safe use of nanopesticides, it is essential to develop nanoformulations that are based on green nanotechnology and that offer low cost, simple procedures, and controlled-release features. In conclusion, the use of smart delivery nanopesticides is highly promising as an effective tool for sustainable agricultural development.

## Data Availability

Not applicable.

## Abbreviations

EE: encapsulation efficiency
GO: graphene oxide
HMS: hollow mesoporous silica
Hy: hymexazol
NBA: 3-nitro-4-bromomethylbenzoic acid
NIR: near-infrared
PDA: polydopamine
PEG: polyethylene glycol
PEI: pendimethalin
PEO: polyoxyethylene
Se: selenium
WP[5]A: carboxylatopillar[5]arene ammonium
2,4-d: dichlorophenoxyacetic acid
